# Arginine Gemini-Based Surfactants for Antimicrobial and Antibiofilm Applications: Molecular Interactions, Skin-Related Anti-Enzymatic Activity and Cytotoxicity

**DOI:** 10.3390/molecules28186570

**Published:** 2023-09-11

**Authors:** Francisco Fábio Oliveira de Sousa, Aurora Pinazo, Zakaria Hafidi, María Teresa García, Elena Bautista, Maria del Carmen Moran, Lourdes Pérez

**Affiliations:** 1 Laboratory of Quality Control, Bromatology & Microbiology, Department of Biological & Health Sciences, School of Pharmacy, Federal University of Amapá, Rodovia Juscelino Kubitscheck, km 02, Macapá 68903-419, Brazil; 2 Department of Surfactants and Nanobiotechnology, Instituto de Química Avanzada de Cataluña, Centro Superior de Investigaciones Científicas IQAC-CSIC, 08035 Barcelona, Spain; aurora.pinazo@iqac.csic.es (A.P.); zhatnt@cid.csic.es (Z.H.); teresa.garcia@iqac.csic.es (M.T.G.); elena.bautista@iqac.csic.es (E.B.); 3 Secció de Fisiologia, Departament de Bioquímica i Fisiologia, Facultat de Farmàcia i Ciències de l’Alimentació, Universitat de Barcelona, Avda. Joan XXIII 27-31, 08028 Barcelona, Spain; mcmoranb@ub.edu; 4 Institut de Nanociència i Nanotecnologia—IN2UB, Universitat de Barcelona, Avda. Diagonal 645, 08028 Barcelona, Spain

**Keywords:** gemini arginine-based surfactants, antimicrobial activity, biofilm inhibition, biofilm eradication, Langmuir monolayers, enzymatic activity, molecular docking, cytotoxicity

## Abstract

The antimicrobial and antibiofilm properties of arginine-based surfactants have been evaluated. These two biological properties depend on both the alkyl chain length and the spacer chain nature. These gemini surfactants exhibit good activity against a wide range of bacteria, including some problematic resistant microorganisms such us methicillin-resistant *Staphylococcus aureus* (MRSA) and *Pseudomonas aeruginosa*. Moreover, surfactants with a C_10_ alkyl chain and C_3_ spacer inhibit the (MRSA) and *Pseudomonas aeruginosa* biofilm formation at concentrations as low as 8 µg/mL and are able to eradicate established biofilms of these two bacteria at 32 µg/mL. The inhibitory activities of the surfactants over key enzymes enrolled in the skin repairing processes (collagenase, elastase and hyaluronidase) were evaluated. They exhibited moderate anti-collagenase activity while the activity of hyaluronidase was boosted by the presence of these surfactants. These biological properties render these gemini arginine-based surfactants as perfect promising candidates for pharmaceutical and biological properties.

## 1. Introduction

Gemini surfactants are composed of at least two hydrophobic alkyl chains and two ionic or polar heads linked by a spacer chain [1]. Due to their interesting properties, these compounds have attracted great interest. Compared with their monocatenary homologues, they can be many orders of magnitude more surface active, display very low critical micelle concentration (cmc) and high solubilization properties. Interestingly, the biological and physio-chemical properties of these surfactants can be fine-tuned for diverse applications by modifying some of their relevant structural features: hydrophobic moiety, polar head or spacer chain. Cationic gemini surfactants are of special consideration owing to their good antimicrobial properties; in general, these dimeric surfactants exhibit a stronger antimicrobial activity than their monomeric homologues [2,3].

Nowadays, the rapid emergence of drug-resistant bacteria and fungi are a major concern for human health. The World Health Organization recognizes that infectious diseases produced by resistant microorganisms are one of the top threats to worldwide health [4]. Many pathogenic bacteria are able to form biofilms: complex communities that exist in an extracellular matrix. Bacterial cells in biofilms are 100–1000 times more resistant to standard antimicrobials than planktonic ones. In recent years, biofilms have become of an extreme clinical importance as they are associated with many persistent and chronic bacterial infections [5]. It is estimated that about 65% of all bacterial infections are associated with bacterial biofilms [6]. Bacterial pathogens such as *Pseudomonas aeruginosa* can cause devastating chronic biofilm infections in immune-compromised hosts, in patients with Cystic Fibrosis (CF) and on the surface of medical devices and burn wounds [7]. Similarly, biofilm formation by MRSA and methicillin-sensitive *S. aureus* (MSSA) strains is considered an important virulence factor influencing its persistence in both the environment and the host organism, showing increased resistance against antimicrobial treatment and causing a significant public health problem [8]. Considering this situation, it is urgent to develop novel antimicrobial compounds with a lower propensity for bacterial resistance. Moreover, it is critical that these new antimicrobials can prevent, disperse and treat bacterial biofilms.

**Figure 1:**
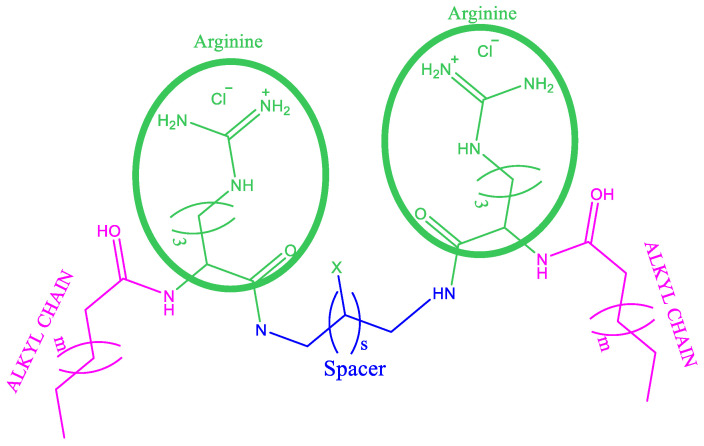
Illustration of arginine-based surfactants. C_3_(OA)_2_ X = H, S = 1, m = 4; C_3_(CA)_2_ X = H, s = 1, m = 6; C_3_(LA)_2_ X = H, S = 1, m = 8; C_3_(LA)_2_OH, X = OH, S = 1, m = 8; C_6_(LA)_2_ X = H, S = 4, m = 8; C_9_(LA)_2_ X = H, S = 7, m = 8.

Due to their interesting properties, cationic gemini surfactants can be an interesting alternative to develop new antimicrobial and antibiofilm strategies that can help to reduce the development of resistant pathogenic bacteria. Gemini surfactants with quaternary ammonium groups in the polar head, bis-QACs, have been the most widely studied geminis [9,10,11]. These compounds show very good antimicrobial activity against a wide spectrum of microorganisms, inhibit the growth of bacterial biofilms and are effective in eradicating mature biofilms [12]. Moreover, their mode of action mainly involves the disintegration of bacterial membrane, which makes the development of resistant bacteria difficult [13]. However, due to their high chemical stability and low biodegradability, these surfactants could accumulate in the environment, giving rise to serious negative ecological impacts and the proliferation of resistant bacteria [14]. Seeking to reduce these drawbacks, several gemini surfactants based on natural amino acids have been prepared [15]. Years ago, our group synthesized cationic arginine-based gemini surfactants. They consist of two N-acyl-arginine residues linked by a polymethylene spacer chain of different lengths (Figure 1). These geminis combine the special physicochemical properties of conventional bis-QACs with the sustainability and biocompatibility of amino acid-based surfactants. They can be prepared using renewable starting materials such as amino acids and fatty acids, show excellent surface activity and very low cmc values, are effective against a wide spectrum of bacteria, show good biodegradability and have a lower cytotoxicity than their bis-QACs homologues [15].

The purpose of this work is to determine how the nature of the alkyl chain, as well as the spacer chain, (Figure 1) influences the antimicrobial and antibiofilm activity of these bis-Args against several problematic pathogenic bacteria, such as MRSA and *P. aeruginosa*. Interactions of most surface-active compounds with membrane models were investigated with Langmuir monolayers and their enzymatic activity against key enzymes enrolled in the skin-repairing processes were evaluated. Finally, the cytotoxicity over immortal human keratinocytes (HaCaT) and squamous carcinoma cells (A431) was assessed.

## 2. Result and Discussion

### 2.1. Ionization State

All the surfactants studied in this work have a cationic charge on the guanidine group of the arginine. In an aqueous solution, this group behaves like a weak acid; therefore, these surfactants have a weak acidic character and present an acid-base equilibrium with an associated acidity constant that depends on the pH and on a monomer–aggregate equilibrium [16].

In order to determine the ionization state of these gemini surfactants at the pH used to investigate their biological properties, the apparent acid pKa was determined for each of them. The pKap value has been determined by means of a direct titration with NaOH of an aqueous solution of each compound. The surfactant concentrations used were in all cases higher than the minimum aggregation concentration, thus avoiding possible deviations in the pK value due to the monomer–aggregate balance. The pKap values can be estimated from the pH at the semi-equivalence point that is reached when the molar ratio of protonated and non-protonated species is 1:1. In these conditions, we have that the pH = pKa. The results are shown in Table 1.

In all cases, the pKap values are smaller than those described by Fitch [18], as well as those cited in many biochemistry textbooks. The presence of fatty chains in arginine surfactants induces the formation of aggregates in which the positive charges are close to each other. As a result, the repulsion between these charges induces the release of protons, thus increasing the acid character of the compound. Table 1 also contains the reported cmc for these surfactants. The cmc is mainly governed by the hydrophobic content of the molecules. For the same spacer chain, the cmc drastically decreases as the alkyl chain increases. For the same alkyl chain of 12 carbon atoms, the cmc slightly diminishes as the number of the methylene groups in the spacer increases. Unconventional aggregation behavior and two cmcs were inferred from different techniques. The first cmcs, inferred primarily from tension data, are between 0.002 and 0.005 mM. Fluorescence and conductivity experiments indicated a second cmc at concentrations two orders of magnitude higher than that from surface tension (0.3–0.6 mM). The low cmc obtained by surface tension measurements was ascribed to non-globular aggregates with small aggregation numbers larger than two or three, given the nearly constant surface tension above these cmc. The surface tension values at the first cmc were in the same order for all gemini surfactants. In general, compounds with 12 carbon atoms in the alkyl chain have moderate water solubility, very good surface activity and form aggregates at very low concentrations [19]. The C_10_ derivative, C_3_(CA)_2_, has very good water solubility and also reduces water surface tension at very low concentrations.

### 2.2. Antimicrobial Activity

**Figure 2:**
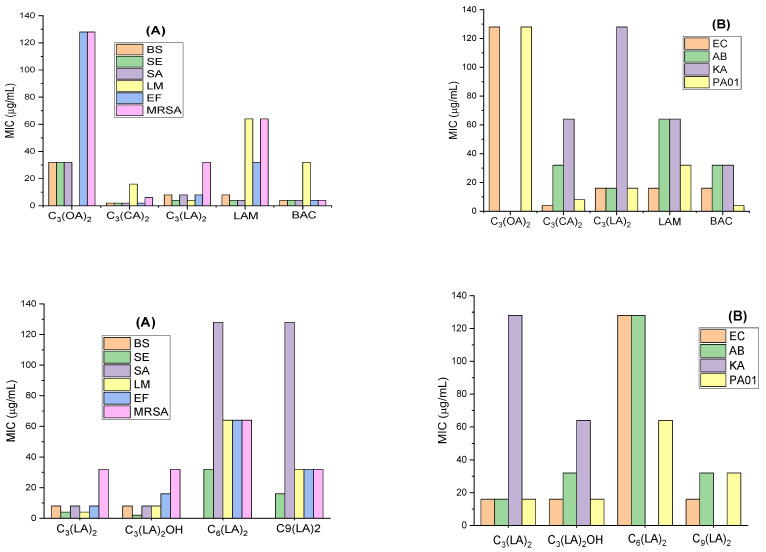
MIC values of the gemini arginine-based surfactants against Gram-positive bacteria (**A**) and Gram-negative bacteria (**B**) *B. subtilis* (BS), *S. epidermidis* (SE), *S. aureus* (SA), *L. monocytogenes* (LM), *E. faecalis* (EF), methicillin resistant *S. aureus* (MRSA), *E. coli* (EC), *A. baumannii* (AB), *K. aerogenes* (KA), *P. aeruginosa* (PAO1).

The minimum inhibitory concentration (MIC; the concentration of gemini surfactant required to completely inhibit the growth of the microorganisms) against representative Gram-positive and Gram-negative bacteria was determined. The Gram-positive bacteria tested include some problematic microorganisms such us *L. monocytogenes*; these bacteria cause listeriosis, a serious disease that has grown considerably in industrialized countries due to the increased ratio of immunosuppressed persons and the extensive use of refrigerated food. We have also tested the effectivity of these surfactants against methicillin-resistant *Staphylococcus aureus* (MRSA), which is known as a major cause of hospital-acquired infections and, at this time, is a remarkable public health problem. Figure 2 shows the MIC values for the gemini surfactants in which the alkyl chain and the spacer chain have been systematically varied. For comparison, this figure also includes the MIC values of a LAM (lauroyl arginine methyl ester) surfactant that can be considered the single-stranded homologue of these geminis and the BAC (benzalkonium ammonium chloride), a quaternary ammonium surfactant widely used as antiseptic.

These gemini surfactants show good antimicrobial activity against a wide spectrum of microorganisms. As expected, the activity depends on their molecular structure, number of carbon atoms in their hydrophobic moiety and in the spacer chain. Our results indicate that these amino acid-based surfactants exhibit a greater effectivity against Gram-positive bacteria. This behavior is common for antimicrobials exhibiting a detergent-like mechanism [20]. The target of these compounds is the bacterial membrane. Given the excellent surface-active properties and the two positive charges present in their structure, it is expected that the mechanism of action of all these bis-Args involves the electrostatic interaction between the two positive charges of the protonated guanidine groups and the negative charges in the bacterial membranes, and then the hydrophobic interaction of the two alkyl chains in the intramembrane region. In fact, it was found that one of these geminis, the C_3_(CA)_2_, and the gemini-lysine surfactants exhibited this mode of action [20]. The bacterial membrane of the Gram-negative bacteria has an external layer mainly composed of peptidoglycan, glicerophospholipids and lipopolisaccharides that hampers the interaction of surfactants with the cellular membranes [21]. As has been mentioned, the mode of action of QACs, widely used biocides, involves the disruption of the bacterial membrane [22], a fundamental target to bacterial survival. As such, in principle, the development of resistant microorganisms to these antimicrobials seems improbable. However, due to their wide use and their low biodegradability, their accumulation in the environment is inevitable. This means that bacterial populations are exposed to sub-lethal doses of these compounds for long periods of time. For this reason, in recent decades, the development of bacterial resistance to QASs and bis-QACs has increased alarmingly [23]. The target of bis-Args is also the bacterial membrane, however, these compounds show good biodegradation levels [24] and, therefore will not accumulate in the environment and, consequently, the development of bacteria resistance to these compounds will be very unlikely.

The bis-Args surfactants with C_10_ and C_12_ alkyl chains and a C_3_ spacer showed better activity than their single counterpart N-lauroyl arginine methyl ester (LAM). Similar results have been already reported for numerous gemini surfactants of the bis-QACs type [25] and amino acid-based gemini amphiphiles [20]. The presence of two positive charges and two alkyl chains facilitates the electrostatic and hydrophobic interactions of these antimicrobials with the bacterial membranes. The pKa values of these compounds indicate that they have two positive charges at a physiological pH, which is a crucial characteristic for damaging bacterial membranes. In this regard, molecular dynamic models proved that arginine is the only natural amino acid residue that remains protonated inside the biological membranes [26]. On the one hand, increasing the cationic charge density of the polar head is one common strategy to improve the antimicrobial efficiency. Haldar et al. [27] prepared cationic surfactants containing one, two and three trimethylammonium head groups and found that the antimicrobial activity increased as the number of cationic groups increased. On the other hand, for the same cationic charge density, double-chain surfactants exhibit better antimicrobial activity than their corresponding single-chain homologues. For example, the 12-s-12 bis-QACs have the same cationic charge density than single-stranded homologue dodecyltrimethylammonium chloride (DTAB); however, the activity of these bis-QACs against *E. coli* and *S. aureus* is 2–5 times higher than that of DTAB [25].

As expected, the antimicrobial activity of these surfactants depends on the alkyl chain length. It is well known that the antimicrobial activity of cationic surfactants does not have a linear dependence with the length of their hydrophobic length and usually exhibits a cut-off effect [25]. This behavior has been also observed for these gemini surfactants. The compound with the shortest alkyl chain, C_3_(OA)_2,_ showed the lowest antimicrobial efficiency (highest MIC values). The alkyl chains of this surfactant are too short and give rise to weak hydrophobic interactions with the bacterial cell membranes. The incorporation of two methylene groups in the hydrophobic chain C_3_(CA)_2_ resulted in an important increase in the antimicrobial activity. A further increase in the alkyl chain C_3_(LA)_2_ produced a slight reduction in their efficacy. These results suggest that for these gemini homologues, the cut-off effect occurs for the C_10_–C_12_ derivatives; however, for the single chain arginine derivatives, the N-acyl arginine methyl esters, the most efficient surfactants for killing bacteria were the C_12_–C_14_ homologues [20]. Usually, the cut-off effect for cationic gemini surfactants occurs at shorter alkyl chains than for their monocatenary homologues. The highest activity of the monocatenary QACs was observed for the C_14_–C_16_ homologues. However, the MIC of the QAC gemini surfactants reached the minimum value for the C_10_–C_12_ derivatives [3]. The same tendency was observed for gemini histidine-based surfactants; in this case, the highest efficiency of the monocaternary homologues was observed for the C_12_–C_14_ homologues, while the C_10_ derivative was the most effective gemini compound [28]. This behavior could be ascribed to the higher hydrophobic character of the gemini surfactants and their extraordinarily low cmc values.

Figure 2 shows the influence of the spacer chain nature. It can be observed that the incorporation of one OH group in the spacer does not significantly affect the antimicrobial activity and the MIC values of C_3_(LA)_2_ and C_3_(LA)_2_OH are similar. However, the elongation of the spacer chain, the C_6_ and C_9_ derivatives, decreases the efficiency of these compounds. It has been observed that the activity of C_3_(LA)_2_ is slightly lower than that of C_3_(CA)_2_, suggesting that the hydrophobic character of this surfactant is in the cut-off limit for antimicrobial activity. Because of that, the C_6_ and C_9_ homologues with higher hydrophobic contents exhibited a lower efficiency than the C_3_ derivative. The research regarding the influence of the spacer in the antimicrobial efficiency is still rather scarce. Zhang et al. found that for the same alkyl chain, the 12-s-12 compounds, the MIC values increased in long spacer chains [29]. It has been also reported that the incorporation of an extra cationic charge in the spacer of bis-QACs improves the antimicrobial efficacy [12].

The antimicrobial activity of BAC is similar to that obtained for C_3_(CA)_2_ and higher than that observed for all C_n_(LA)_2_. BAC is not a pure cationic surfactant; it is a mixture of alkylbenzyldimethylammonium chlorides with alkyl chains of C_8_, C_10_, C_12_, C_14_, C_16_, C_18;_ dodecyl and tetradecyl being the most abundant ones (about 40% C_12_, 50% C_14_). The good biocidal activity of this compound is associated with the mixture of C_12_ and C_14_ alkyl chain derivatives that gives the compound a very good water solubility. However, this compound is irritating to the skin and eyes and toxic to aquatic organisms. Moreover, BAC shows a low biodegradability; consequently, long-term exposure to this disinfectant can result in a change in microbial community and an increased antimicrobial resistance.

Our obtained results indicate that compound C_3_(CA)_2_ was the most active against all tested microorganisms. This surfactant showed very good activity against all Gram-positive and Gram-negative planktonic bacteria, including some problematic microorganisms such as MRSA, *L. monocytogenes* and *P. aeruginosa* bacteria that are commonly resistant to several antimicrobial agents.

#### Antibiofilm Activity

Nowadays, bacterial and fungal biofilms cause more than 65% of microbial infections and are one of the virulence factors promoting the development of resistant microorganisms [6]. The development of a bacterial or fungal biofilm starts with the adhesion of microorganisms to surfaces. Once the biofilm is formed and matured, it is very hard to eliminate due the presence of the polymeric matrix [5]. Therefore, it is crucial to design and synthesize effective antimicrobials capable of inhibiting biofilm formation and eradicating mature biofilms. In this work, we have investigated how the length of the alkyl chain and the nature of the spacer affect the ability of these gemini surfactants to inhibit and disrupt MRSA and *P. aeruginosa* biofilms.

**Figure 3:**
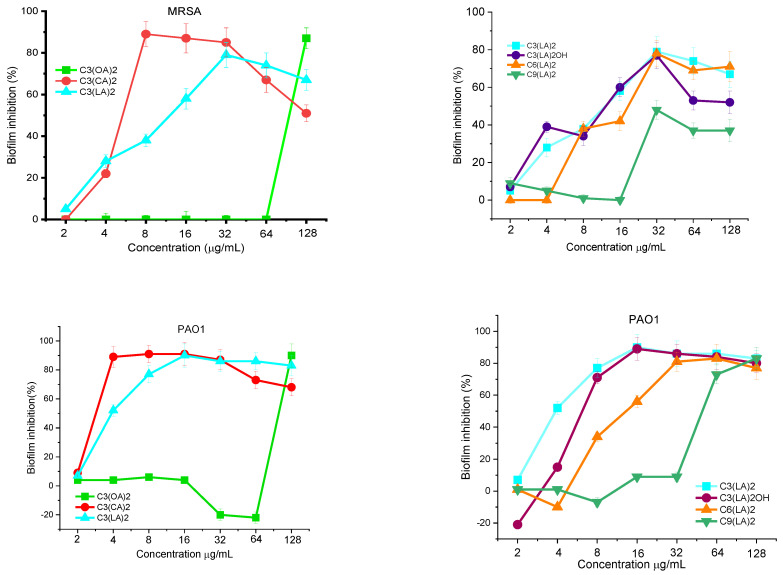
MRSA and PAO1 biofilm inhibition by the investigated arginine gemini surfactants. The percentage of biofilm inhibition was determined by taking into account the biofilm obtained with untreated bacteria (defined as 100%) and that obtained without bacteria (defined as 0%). Data points represent the average (±SD) of 4 replicates.

The ability of MRSA and *P. aeruginosa* to form biofilms on propylene surfaces in the presence of bis-Args was determined. The two bacterial cells were kept in contact with the gemini surfactants at different concentrations from 2 to 128 μg/mL for 24 h. Then, the percentage of biofilm inhibition was determined. Figure 3 shows the effect of the alkyl chain length on the capacity of these compounds to inhibit the growth of MRSA and *P. aeruginosa* biofilms. The best activity was found for the compound with C_10_ alkyl chains; this compound inhibits around 90% of the biofilm formation of these two bacteria at very low concentrations of around 4–8 μg/mL. By increasing the alkyl chain, the antibiofilm activity slightly decreased and the C_3_(LA)_2_ is able to inhibit the growth of both microorganisms at 32 μg/mL. A drastic decrease in the antibiofilm effectiveness was found for the gemini with the shortest alkyl chain, C_3_(OA)_2_; in this case, the biofilm inhibition is only reached at 128 μg/mL.

Figure 3 shows how the nature of the spacer chain affects the biofilm formation in presence of these surfactants. The results indicate that for a C_12_ alkyl chain, the antibiofilm activity against MRSA decreases as the hydrophobicity of the molecule increases. The PAO1 biofilm inhibition follows the same behavior and the inhibition occurs at similar gemini concentrations.

All these results are consistent with the tendencies observed in the MIC values. The biofilm inhibition can be produced by two mechanisms: the compounds coat the polystyrene plate via hydrophobic interactions or/and the surfactants kill the planktonic bacteria in the solution. In this case, the inhibition occurs around the MIC values. These results suggest that the main mechanism involved in this inhibition is the interaction of these gemini with the planktonic cells. In this regard, it has been observed that bacteria die rapidly when kept in contact with these gemini surfactants at concentrations ≥MIC, so they cannot form biofilms.

**Figure 4:**
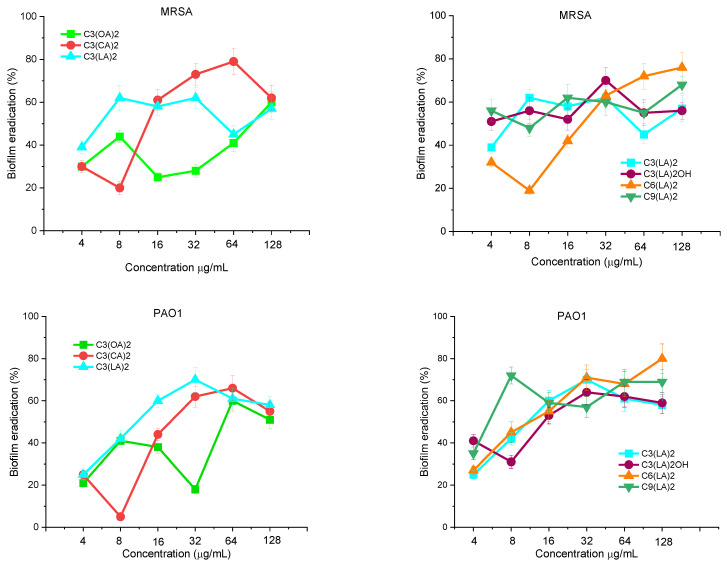
MRSA and PAO1 biofilm eradication with bis(Args) surfactants. Data points represent the average (±SD) of 4 replicates.

The ability of these geminis to disperse and eradicate biofilms once formed on the propylene surfaces of the microtiter plates was evaluated using the same bacterial strains. Figure 4 shows the percentage of biofilms eradicated by the tested compounds at concentrations ranging from 4 to 128 μg/mL. The susceptibility of both biofilms against the tested surfactants was similar. At a low concentration of 32 μg/mL, C_3_(CA)_2_ and C_3_(LA)_2_ were able to eradicate around 60–70% of preformed biofilm; however, at this concentration, the lowest hydrophobic compound, C_3_(OA)_2,_ eradicated less than 30% of biofilm. At high concentrations, the eradication percentage is similar for all alkyl chains. Figure 4 shows the spacer chain nature. In this case, at low concentrations, there is not a clear dependence between the antibiofilm properties and the spacer; however, at a high concentration, the best effectivity was obtained for the longer spacer chains.

The bis-Args concentration required to eradicate MRSA and *P. aeruginosa* biofilms is higher than that required to inhibit their formation. It has already been observed that usually it is usually more difficult to eradicate biofilms than to inhibit their formation [30]. At the MIC concentration, cationic surfactants usually inhibit biofilm formation because they kill bacteria. However, at this concentration, these compounds are not active against bacterial cells in mature biofilms [31]. In this case, the mechanism of action of antimicrobials involves two steps: the disruption of the extracellular polymeric matrix by electrostatic interactions and then the elimination of the dispersed bacteria.

It has been described that monocatenary cationic surfactants based on quaternary ammonium groups show remarkable antibiofilm activities [30,32,33]. The antibiofilm activity of amino acid-based surfactants has been scarcely reported. Tac-seung et al. [34] found that N-lauroyl arginine ethyl effectively eliminated biofilms from reverse osmosis membranes and Rodriguez Almeida et al. [35] described small cationic peptide amphiphiles that reduced MRSA biofilm formation up to 69% at MIC (8 μg/mL) and up to 90% at 2ϗMIC, whereas Pinazo et al. demonstrated that single-chain arginine surfactants efficiently eradicate MRSA biofilms at higher values than that observed for these gemini arginine surfactants, at 32 μg/mL. The antibiofilm activity of cationic gemini surfactants is still poorly known. Jenning et al. [12] found that the presence of two or three cationic charges on the polar head and two-alkyl-chain, bis(QACs) notably enhanced the biofilm-eradicating properties of quaternary ammonium surfactants against *S. aureus* and *E. faecalis*. The MBEC values obtained for the tested bis(QACs) ranged from 50 to 100 μM. Kozirog et al. found that a 12-6-12 gemini surfactant effectively prevented *A. lannensis* biofilm growth in propylene surfaces and is also effective at eradicating biofilm in this bacteria [31], and Oblak et al. reported that gemini quaternary ammonium salts inhibited the adhesion of *S. epidermis* to a polystyrene surface and eradicated biofilm formed by PA01; they found that the best antibiofilm activity was obtained for C_12_ derivatives. At 20 μM, these gemini destroyed about 50% of the biofilm [36]. There are very few reports in the literature describing the antibiofilm activity of gemini surfactants based on amino acids. Alanine-based gemini quaternary ammonium salts with two carbon spacer groups and C_10_–C_12_ carbon alkyl chains (chlorides and bromides) dislodged the biofilms of *P. aeruginosa* and *S. epidermidis* and effectively reduced microbial adhesion by coating the polystyrene and silicone surfaces [37].

### 2.3. Surface Pressure Molecular Area Isotherms and Elastic Modulus

Aimed at establishing models of surfactant–bacteria interactions, we have studied the interactions between three arginine-based surfactants and three different phospholipids. The studied surfactants included three gemini surfactants, namely C_3_(LA)_2,_ C_6_(LA)_2_ and C_9_(LA)_2_. The study of the three geminal surfactants shall allow us to establish the influence of the spacer length on the interactions with phospholipids.

The phospholipids considered were DPPC (Dipalmitoylphosphatidylcholine), DPPE (Dipalmitoylphosphatidylethanolamine) and DPPG (dipalmitoylphosphatidylglycerol). DPPC and DPPG were selected because they are the main constituents of the bacterial membrane’s phospholipids, while DPPE is the main phospholipid in erythrocytes.

The surface pressure molecular area π-A isotherms (Langmuir isotherms) can be used as a tool to obtain information of the interaction between surfactants and phospholipids placed in an air–liquid interface. The shape of the compression isotherm shows the extent to which the surfactant is forced into the bulk subphase. If the surfactant is completely desorbed as the film is compressed, the resulting isotherm would match that of pure phospholipid. Thus, any deviation from this behavior can be attributed to an incomplete desorption of the surfactant [38].

#### 2.3.1. Langmuir Isotherms

**Figure 5:**
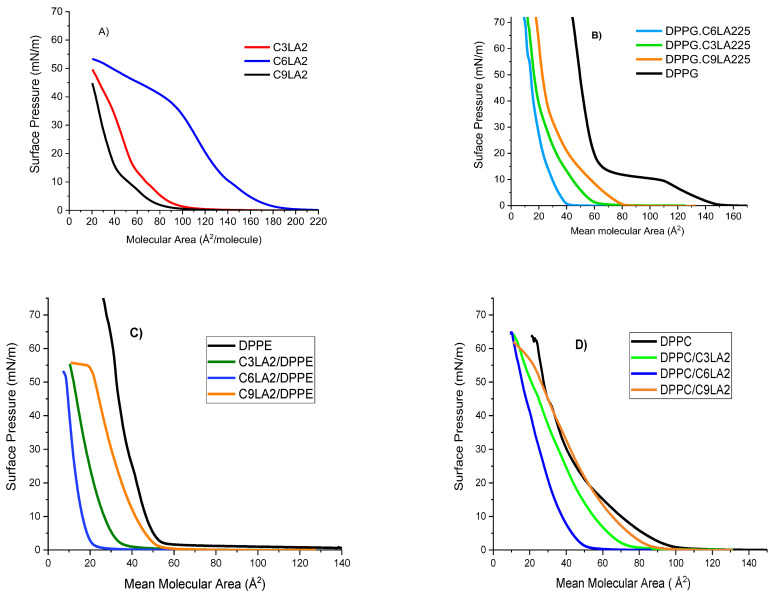
π-A isotherms for (**A**) pure phospholipids and pure arginine gemini surfactants, (**B**) DPPG/surfactant mixtures, (**C**) DPPE/surfactant mixtures and (**D**) DPPC/surfactant mixtures.

The π-A isotherms for pure phospholipids and pure arginine gemini surfactants, as well as their mixtures, are shown in Figure 5. Due to this redissolution, the registered areas per molecule of gemini surfactants are smaller than expected. The results were similar to those reported in previous work [39].

First, we considered mixtures of gemini surfactants with DPPC. The profile of their isotherms corresponds to surfactants with partial solubility. Under compression, part of the molecules located at the interface redissolve either as aggregates or as monomers.

As mentioned, isotherms of pure phospholipid are used as references; hence, they are included within the phospholipid gemini surfactant π-A plots (Figure 5). The isotherm of pure DPPC fully agrees with that reported in [40]. It is noticed that mixtures of isotherms appear at areas lower than those of the pure DPPC. The plots shift toward smaller areas, according to the series

DPPC/C_9_(LA)_2_ < DPPC/C_3_(LA)_2_ < DPPC/C_6_(LA)_2_

These shifts suggest two facts. First, both the length and flexibility of the spacer chain have an influence on the interactions with DPPC. Second, we can expect that in the mixed monolayer, the two components interact to form mixed aggregates which have a solubility higher than pure DPPC, favoring the phospholipid to move towards the bulk phase. C_9_(LA)_2_ is the surfactant with the largest spacer chain. The mixture C_9_(LA)_2_ -DPPC shows an isotherm almost parallel to that of the DPPC, which collapses at a molecular area slightly lower than that of the DPPC. Since C_9_(LA)_2_ has a large and flexible spacer chain, the interaction is favored, the compound remains in the monolayer and the formation and solubilization of mixed aggregates is not extensive.

The surfactant C_3_(LA)_2_ has a short spacer chain; thus, when mixed with DPPC, forms mixed aggregates that, under compression, move to the bulk phase and results in a plot shift larger than that of C_9_(LA)_2_. The isotherm plots of C_3_LA-DPPC and C_9_(LA)_2_-DPPC are very close to each other. This small shift suggests that both surfactants interact with DPPC through one arginine group. The six carbon atoms included in the spacer chain of C_6_(LA)_2_ conform to rigid spatial structures that can interact through its two arginine groups, with the DPPC forming mixed aggregates that move to the bulk phase, resulting in the largest plot shift.

Mixtures of gemini surfactants with DPPE are also shown in Figure 5. The pure DPPE isotherm agrees with as is reported in the literature [40]. The isotherms of three mixtures considered were shifted toward lower areas, according to the series

DPPE/C_9_(LA)_2_ < DPPE/C_3_(LA)_2_ < DPPE/C_6_(LA)_2_

A rationale for this behavior is that interactions between one of the guanidine groups of the gemini surfactants and DPPE could lead to the formation of cationic aggregates which are soluble in the subphase.

Clearly, isotherms of mixtures with DPPC are different from those with DPPE. Although both phospholipids are zwitterionic, DPPC carries in its polar head a quaternary ammonium group, while DPPE carries an ammonium group. Differences in the effects that phospholipids induce in the mixtures should be mainly attributed to the fact that the DPPC has three methyl groups in its polar head, thus showing a large hydrophobic character.

The interactions of gemini surfactants with an anionic phospholipid, DPPG, have been also studied. Plots of the DPPG/arginine mixture isotherms are shown in Figure 5. Since DPPG has an anionic charge in its polar head, interactions with arginine cationic surfactants are strong and result in conformational changes in the molecules at the interface, but this does not imply their dissolution in the bulk. The presence of a significant number of molecules at the interface gives rise to high values in collapse pressure.

Isotherms for three mixtures shifted to lower areas according the series

DPPG/C_9_(LA)_2_ < DPPG/C_3_(LA)_2_ < DPPG/C_6_(LA)_2_

#### 2.3.2. Mechanical Properties of the Mixed Monolayers

The rigidity of a monolayer under pressure depends on the interfacial cohesive forces and can be measured using the elastic modulus: a mechanical concept defined as

E = −A (δπ/δA)

where A is the area and π is the pressure exerted on A. The higher the elastic modulus, the greater the material rigidity. Therefore, once the π–A isotherms are known, the elastic modulus E associated with the monolayer can be calculated. Clearly, as the E value associated with the monolayer grows, the monolayer elasticity decreases due to increasing interfacial cohesive forces. Values of E in the range 100–200 m/mN are typical of condensed phases [41].

**Figure 6:**
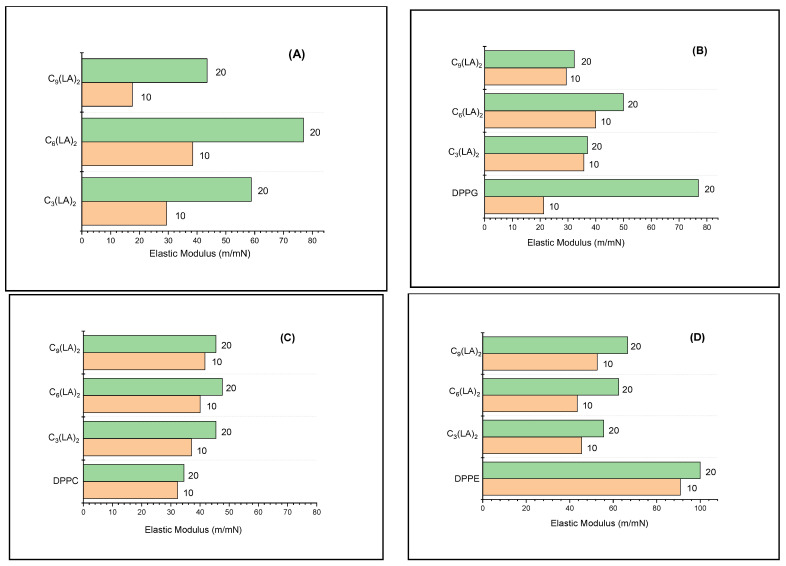
Surface compressional modulus (E) of pure surfactants (**A**), DPPG/surfactant mixtures (**B**), DPPC/surfactant mixtures (**C**) and DPPE/surfactant mixtures (**D**). E values calculated at π = 10 and π = 20.

To avoid secondary effects, measurements of E have been carried out at π = 10 mN/m and π = 20 mN/m when the monolayers studied are in an expanded phase and structural differences of the mixture components are clearly identified. Figure 6 shows E values measured for arginine surfactants and their mixtures with DPPC, DPPE and DPPG. Under compression, monolayers of pure gemini arginine surfactants form expanded gas and liquid phases. At π = 10 mN/m, E values correspond to typical values of expanded monolayers for the four surfactants studied. At π = 20 mN/m, the maximum observed value is 80 mN/m, which corresponds to the compound C_6_(LA)_2_. This value is close to the elasticity values presented by the condensed monolayers [39]. The E values of the mixtures of these surfactants with DPPC are all around 40 m/mN, which is slightly lower than those of the surfactants alone. Therefore, the mixed monolayer is less compacted, more disordered and more fluid than the pure surfactant monolayer. When compared to the E values of pure DPPC, a slight increase in the mixtures was observed. These values indicate that arginine and DPPC interact to form a mixed monolayer. The values of the modulus of elasticity obtained for the DPPC agree with those previously reported [42].

Mixtures of C_3_(LA)_2_ with DPPC showed E values similar to mixtures with DPPE, while for C_6_(LA)_2_ and C_9_(LA)_2,_ the E values were slightly higher. The E values for DPPE alone are close to 100 m/mN, thus reflecting the formation of a condensed phase monolayer.

These results show that the interactions of arginine-derived surfactants with amphoteric phospholipids depend, on the one hand, on the hydrophobic component of the surfactant and, on the other hand, on the nature of the cationic charge on the polar head of the phospholipid.

The E values of the mixtures with DPPG are lower than those obtained with DPPC and DPPE. The negative charge on the polar head of DPPG forms an ion pair with the positive charge of the guanidino group present in arginine-derived surfactants. The values obtained for C_6_(LA)_2_ suggest that it is only one guanidino group of a molecule that interacts with DPPG. C_3_(LA)_2_ and C_6_(LA)_2_ show smaller values of E, thus reflecting a greater disorder in the monolayer, probably because the guanidino groups that interact belong to adjacent molecules.

In general, interactions of cationic surfactants derived from the amino acid arginine with simple membrane models strongly depend on the structure of the surfactants. Modifying the structure of the surfactant, either in the polar head or in the hydrophobic part, entails changes in the interactions. The gemini structure surfactants studied here have two cationic charges so they can form aggregates via ion pairs, which are then solubilized in the sub phase under compression. As a result, isotherms are shifted towards smaller areas. However, there are other factors that influence the surfactant membrane model’s interactions. Mixed monolayers of gemini surfactants derived from the amino acid Lysine [43] as well as the amino acid Histidine [28] carry two cationic charges on the polar head; however, isotherms of mixtures of each surfactant with phospholipids are displaced towards larger areas with respect to those of the phospholipid alone. This difference can be attributed to the fact that the polar heads of both Lysine and Histidine are more hydrophobic than the polar head of Arginine, resulting in an increase in the overall hydrophobic character, consequently having a significant influence on the interactions with phospholipids.

The number of fatty chains and their length are also key structural factors in surfactant–phospholipids interactions. Synergy is observed in interactions of surfactants with two fatty chains mimetic to a phospholipid [44]; moreover, under compression, stable monolayers are formed. However, the stability of monolayers formed by surfactants derived from arginine which have just one fatty chain [45] is clearly smaller, according to the corresponding values of the elastic module measured.

### 2.4. Anti-Enzymatic Inhibitory Activities

Several proteolytic enzymes are involved in the skin- and connective tissue-repairing mechanisms. For instance, collagenase and elastase are responsible for the degradation of collagen and elastin fibers in the extracellular matrix [46,47]. Controlling their activity plays a key role in skin aging, while the modulation between collagen production and degradation is also important to ensure adequate and shorter wound healing [48,49]. Hyaluronidase is involved in the inflammatory activity and more importantly in the degradation of hyaluronic acid, which is an important dermic filler [50]. In view of these characteristics, the effect of the surfactants in the inhibition of these enzymes was evaluated to explore the potential applications of these compounds.

**Figure 7:**
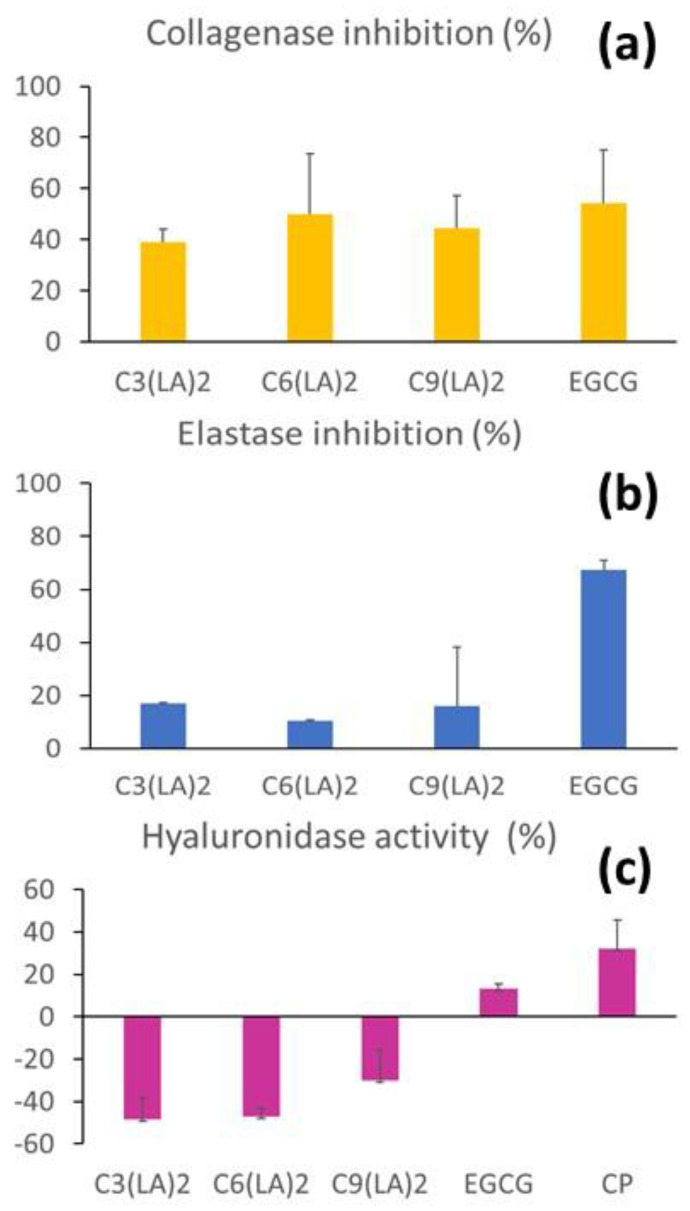
Inhibition of collagenase (**a**), elastase (**b**) and hyaluronidase (**c**) by arginine-based surfactants. C3: C_3_(LA)_2_, C6: C_6_(LA)_2_ and C9: C_9_(LA)_2._ EGCG: epigalocatequin-3-galate. CP: hyaluronidase enzyme.

The inhibitory activities of the surfactants over key enzymes enrolled in the skin repairing processes were evaluated. C_6_(LA)_2_, immediately followed by C_9_(LA)_2_ and C_3_(LA)_2,_ presented moderate anti-collagenase activity (Figure 7a), ranging from approximately 40 to 50%, similar to that found for the positive control EGCG (54.2%). Therefore, the surfactants were found to moderately inhibit the enzyme collagenase. These molecules can be important during the second phase of wound healing to regulate the deposition of proteins during the fibroblast proliferation phase, promoting its modelling. This is also another important aspect to be explored in alternative skin care applications.

The inhibition of the enzyme elastase was far more discrete. C_3_(LA)_2_, C_6_(LA)_2_ and C_9_(LA)_2_ presented anti-elastase activity limited to 17, 10.5 and 15.9%, respectively. In contrast, EGCG presented 67.3% of inhibition (Figure 7b). Therefore, the surfactants did not affect the activity of this enzyme.

Hyaluronidase reverse depolymerizes hyaluronic acid found in the cementum around the cells of the connective tissue. Unlike the previous enzymes, the activity of hyaluronidase was boosted by the presence of these surfactants, demonstrated by the negative values observed. C_3_(LA)_2_, C_6_(LA)_2_ and C_9_(LA)_2_ increased the enzymatic activity by 48.3, 47.2 and 29.9%, respectively (Figure 7c). EGCG presented an inhibitory activity of 13%, while the enzyme control resulted in 32% inhibition. Therefore, the surfactants would contribute synergically with the enzyme hyaluronidase for the removal of hyaluronic acid in specific cases, such as when aesthetic procedures need to be corrected and in special clinical conditions when tissues are formed inadequately and need to be molded. This enzyme also increases the absorption and reduces the pain caused by subcutaneous or intramuscular administration of liquids, enhances the absorption of extravasated liquids and blood on the tissues and improves the efficacy of local anesthesia. In this case, the surfactants could also be useful as an adjuvant to the parenteral administration of drugs, also considering its solubilization and wettability properties.

Hence, the anti-enzymatic activities were identified to support the use of the surfactants in the treatment of specific skin disorders. While collagenase and elastase inhibition may be helpful in maintaining and stimulating the resistance and elasticity of the skin and also the skin repairing, the increased hyaluronidase activity found may be useful in the connective tissues or skin remodeling, for instance, treating hypertrophic and keloid lesions, based on their anti-inflammatory effect.

### 2.5. Molecular Docking Results

Molecular docking is a computational tool capable of identifying several connection sequences and predicting the binding affinity of new compounds at the target receptor binding sites. Molecular docking simulations were carried out to understand the observed enzymatic activities of the studied surfactants and to shed light on the binding modes between docked ligands and enzymatic targets.

**Figure 8:**
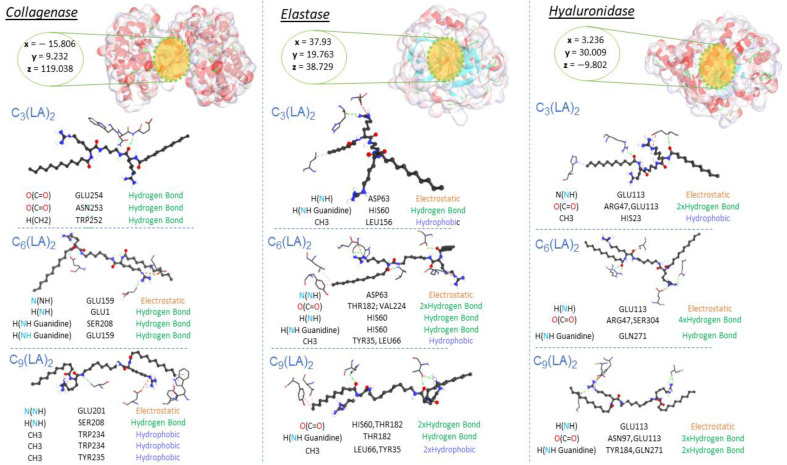
Interaction modes of arginine-based surfactants C_3_(LA)_2_, C_6_(LA)_2_ and C_9_(LA)_2_ in the active sites on the surface of Elastase (PDB ID: 1ELE), Collagenase (PDB ID: 7ESI) and Hyaluronidase (PDB ID: 1FCV).

**Figure 9:**
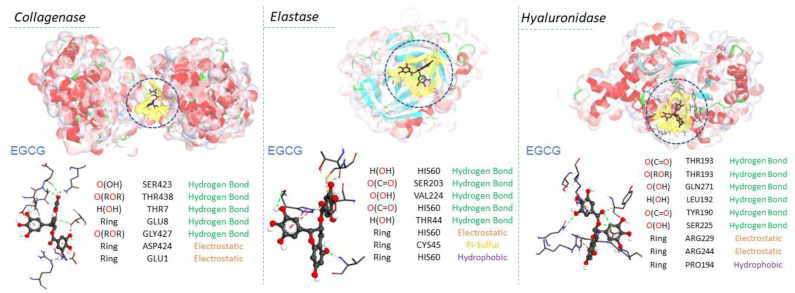
Interaction modes of EGCG: epigalocatequin-3-galate in the active sites on the surface of Elastase (PDB ID: 1ELE), Collagenase (PDB ID: 7ESI) and Hyaluronidase (PDB ID: 1FCV).

The likeliest docked positions of surfactants and EGCG ligands with the best binding affinity for ligand complexes in the active site of the targeted receptors are shown in Figure 8 and Figure 9. All surfactants showed a good affinity to the receptors pocket (Table 2, Table 3 and Table 4), with free energy binding values between −10 and −12.8 kcal/mol for surfactants and between −9.2 and −10.9 kcal/mol for EGCG. Three types of interaction were found in the modes of action for all ligands: hydrogen, electrostatic and hydrophobic interactions, with the exception of a Pi-sulfide type interaction, which was observed in the interaction mode of EGCG against the enzyme elastase.

Regarding collagenase and elastase, the experimental part showed that the EGCG presented a higher inhibition rate (54%) than the surfactants (from 40% to 50%). This aspect corroborates the high affinity of EGCG with these two enzymes in molecular docking. Based on their modes of interaction (Figure 8 and Figure 9), EGCG shows more hydrogen-type interactions compared to surfactants, due to the existence of more hydroxyl groups on its molecular structure, contributing to the proton labile of the OH group to interact with the residues of the enzyme collagenase and elastase. The same aspect was also observed for hyaluronidase in molecular docking, i.e., the two types of molecules, EGCG and surfactants, show in their interaction modes different bonding-types against many residues in the hyaluronidase structure. Unlike the other two enzymes, the addition of surfactants increased the activity of hyaluronidase, as evidenced by the negative values found. Hyaluronidase is a naturally occurring enzyme capable of the local degradation of hyaluronic acid [51]. Hyaluronidase hydrolyzes hyaluronic acid by splitting the bond between the C_1_ of an N-acetyl-glucosamine moiety and the C_4_ of a glucuronic acid moiety [52]. The surfactants C_3_(LA)_2_, C_6_(LA)_2_ and C_9_(LA)_2_ enhanced the enzymatic activity by 48.3, 47.2 and 29.9%, respectively. EGCG presented a 13% inhibitory activity, while the enzyme control had a 32% inhibition. The experimental data indicate that the surfactants enhanced the degradation of hyaluronic acid, enhancing the enzymatic activity. Two hypotheses are possible: either the surfactants hyaluronidase interactions made the enzyme more active or the presence of surfactants made the hyaluronic acid more reactive by solubilizing or exposing the enzymatic target found in the N-acetyl-glucosamine moiety. Unlike the surfactants, EGCG shows hydrogen-type interactions over hyaluronidase (Figure 9). The presence of OH groups on its structure promotes many hydrogen-type interactions. Therefore, this results in a greater affinity, competitively, over the enzyme compared to hyaluronic acid, which makes it less likely to be degraded in the presence of EGCG molecules. In contrast, the surfactants also show a limited hydrogen-type interaction. The molecular structure of hyaluronic acid has a lot of carbonyls and some hydroxyl groups, which can favor the affinity of hyaluronidase to its substrate over the surfactants. This difference may be one of the reasons why, in the presence of surfactants, hyaluronidase becomes more active, resulting in a more extended degradation of hyaluronic acid.

### 2.6. Cytotoxicity

Although these surfactants were found to be very potent in the biological activities tested, some limitations can be found when they are incorporated together with other adjuvants. Moreover, they are commonly associated with the most irritative category of surfactants. Recently, we have demonstrated that nanoencapsulation was a suitable strategy to reduce the hemolytic activity of these compounds, leaving the antimicrobial activities unaltered [39]. In view of these characteristics, in this work, the cytotoxicity of both free surfactants and surfactants loaded to zein nanoparticles was comparatively evaluated. The arginine-based nanoparticles were obtained and characterized according to our previously published paper [39].

**Figure 10:**
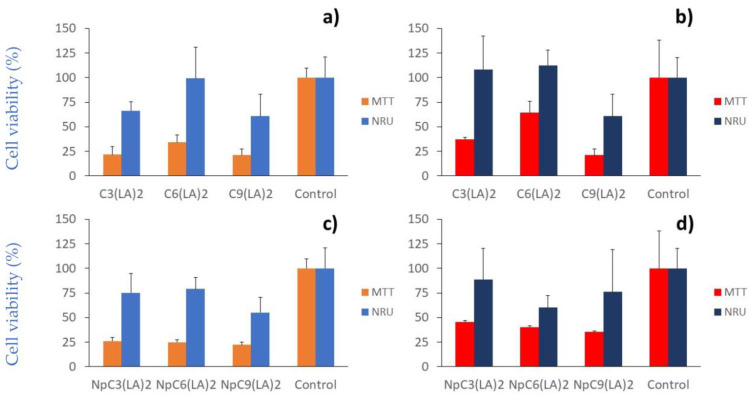
Cytotoxicity of gemini arginine-based surfactants in solution (at 35.6 µg/mL) (**a**,**b**) and encapsulated in zein nanoparticles (at 35.6 µg/mL) (**c**,**d**) over immortal human keratinocytes (HaCaT) and squamous cell carcinoma, respectively.

The cytotoxicity was assessed by the colorimetric methods (MTT and NRU assays) over immortal human keratinocyte (HaCaT) (Figure 10a,b) and squamous cell carcinoma (A431) (Figure 10c,d), comparing the activity of the bulk surfactants and the corresponding nanoparticles with the negative control cells in the absence of any treatment.

The cytotoxic response differed between methods as a consequence of their different interaction with cells. Thus, while the neutral red uptake (NRU) assay relies on the ability of living cells to incorporate and bind neutral red dye though the membranes in lysosomes, the colorimetric assay is based on the reduction in yellow tetrazolium salt (3-(4,5-dimethylthiazol-2-yl)-2,5-diphenyltetrazolium bromide (MTT) to purple formazan crystals by metabolically active cells. The viable cells contain NADPH-dependent oxidoreductase enzymes, which reduce the MTT to formazan. Bearing in mind that the surfactants inhibited some enzymes and also adhere to the cellular membranes, the MTT findings may be limited as the enzymes were not totally available due the inhibition that may have been caused by the molecules assayed. For this reason, the MTT method promoted cell viability values always lower than those obtained in the case of the NRU method.

According to the NRU method, when the cellular response of the surfactants in solution was evaluated, the results demonstrated the same cytotoxicity pattern: C_9_(LA)_2_ > C_3_(LA)_2_ > C_6_(LA)_2_ in both cellular lines (Figure 10a,b). However, while the nanoencapsulation did not markedly alter the cytotoxicity of the surfactants over HaCaT cells (Figure 10c), this procedure seems to enhance the cytotoxicity over A431 squamous carcinoma cells (Figure 10d). Therefore, they were more selective over the cells than the surfactants in the solution. The HaCaT cells remained stable after contact with C_6_(LA)_2_ with a cellular viability of 99.3%, while the viability was reduced to 66.2 and 60.7% after treatment with C_3_(LA)_2_ and C_9_(LA)_2_, respectively (Figure 10a). The nanoparticles of C_6_(LA)_2_ presented a viability of 79.0% over HaCaT cells, immediately followed by C_3_(LA)_2_ (75.2%) and C_9_(LA)_2_ (55.0%), although without statistical significance (*p* > 0.05). Over the A431 cell line, C_9_(LA)_2_ maintained its cytotoxicity with a viability of 60.7%, in contrast to C_3_(LA)_2_ and C_6_(LA)_2,_ which did not cause any apparent cytotoxicity (Figure 10c). In contrast, the nanoparticles hindered a more cytotoxic effect over the A431 cells with the highest cytotoxicity caused by C_6_(LA)_2_ (ca. 40%), followed by C_9_(LA)_2_ (24%) and C_3_(LA)_2_ (12%), without statistical significance (*p* > 0.05) between the nanoparticles (Figure 10d). In addition, C_6_(LA)_2_ nanoparticles were more cytotoxic than the bulk surfactant solution (*p* < 0.05) over A431 cells (Figure 10b,d).

The biomedical applications of these gemini surfactants depend on their ability to selectively kill bacteria without toxic effects on mammalian cells. This selectivity is determined by the IC_50_/MIC ratio, named the therapeutic index (TI). The TI of these surfactants depends on the bacteria strain and on the end point method used to evaluate the cytotoxicity. Considering the NRU method and the HaCaT cell line, it can be assumed that the IC_50_ of all surfactants is higher than 35.6 µg/mL. Then, the TI for the C_3_(LA)_2_ against the Gram-positive bacteria and *E. coli* and PA01 is higher than one.

From the obtained results, it can be assessed that the C_3_(LA)_2_ surfactant is the safest C_12_ derivative to be used in applications that require contact with HaCaT cells. Interestingly, C_3_(LA)_2_ has antimicrobial activity at concentrations below that, producing toxicity against HaCaT cells (IC_50_ is always higher than the highest tested concentration, i.e., 35.6 µg/mL).

## 3. Materials and Methods

### 3.1. Materials

All solvents were reagent grade and were used without further purification. DPPC, DPPE and DPPG phospholipids were purchased from Sigma-Aldrich (Merck Life Science, Madrid, Spain). Arginine, arginine methyl ester and BAC were purchased from Sigma-Aldrich. Fatty acids and diamides were obtained from Fluka. Mueller Hinton broth (MHB) and agar (MHA) were purchased from Difco Laboratories (USA) and HIMEDIA, respectively. Phosphate Buffer Solution (PBS), sodium hydroxide (NaOH) and HCl were acquired from Merck.

#### Synthesis of Gemini Arginine Surfactants

Gemini arginine surfactants were obtained following the synthetic pathway described in [53]. The synthesis method consisted of three steps:  first, *N*^α^-alkyl-l-nitroarginine was synthetized by acylation of nitroarginine, then, two *N*^α^-alkyl-l-nitroarginine molecules were coupled with α,ω-alkylidenediamide and finally, gemini arginine surfactants were obtained by catalytic hydrogenation. The chloride salts of the gemini compounds were obtained by protonation of the guanidine group with a solution of methanol/HCl. The obtained compounds named as Bis (N^α^- lauroyl arginine) (2- hydroxy- 1,3-propane) diamide (C_3_(LA)_2_ OH), Bis (N^α^- lauroyl arginine 1,3-propane) diamide (C_3_(LA)_2_), Bis (N^α^- lauroyl arginine 1,3-hexane) diamide (C_6_(LA)_2_), Bis (N^α^- lauroyl arginine 1,3-nonane) diamide (C_9_(LA)_2_). The obtained compounds were characterized by ^1^NMR, ^13^CNMR and Mass Spectrometry. Chemical shifts and mass signals agreed with the values previously reported in the literature [53]. LAM (N^α^-Lauroyl-arginine methyl ester) was also synthesized using synthetic procedure reported in [51]; arginine methyl ester was acylated with docecyl chloride in water/acetone at pH = 9.

### 3.2. Determination of pKa

The pKa value of each compound has been determined by means of a direct titration with NaOH of an aqueous solution of each compound. The pH was measured using the pH electrode Thermo Electron Corporation ORION 8103SC and the temperature was controlled by a thermostatic bath, Heto CBN 8-30. The pKa values can be estimated from the pH at the semi-equivalence point, which contains a 1:1 molar ratio of protonated and non-protonated species; at this point, pH = pKap.

### 3.3. Monolayer Isotherms

Monolayer isotherm measurements were carried out using a Langmuir balance (KSV Instruments Minitrough, Espoo, Finland, KSV Nima LB software version 3.7, provided with a Wilhelmy plate). Phospholipids and their mixtures with gemini arginine surfactants were dissolved in hexane/methanol (9:1) at 1 mg/mL concentration. For each measurement, 25 µL were spread over 20 mM Tris-buffer aqueous sub phase using a Hamilton micro syringe. After solvent evaporation, the surface pressure (π)-molecular area (A) isotherms were registered. The compression rate was 20 mm/min. The molecular area was calculated as the total surface divided by the total number of molecules deposited at the surface. The molar phospholipid/surfactant mixture ratio was always 80/20.

### 3.4. Antimicrobial Activity

Antimicrobial tests were carried out against the following microorganisms: *Bacillus subtilis* ATCC 6633, *Staphylococcus epidermidis* ATCC 12228, *Staphylococcus aureus* ATCC 29213, *Listeria monocytogenes* ATCC 15313, *Enterococcus faecalis* ATCC 29212, *Escherichia coli* ATCC 25922, *Acinetobacter baumannii* ATCC 19606, *Klebsiella aerogenes* ATCC 13048, methicillin-resistant *Staphylococcus aureus* ATCC 43300 and *Pseudomonas aeruginosa* PAO1. The antimicrobial activities were determined in vitro on the basis of the minimum inhibitory concentration (MIC) values, defined as the lowest concentration of antimicrobial agent that inhibits the development of visible growth of bacteria after 24 h of incubation at 37 °C. The gemini surfactants tested were dissolved in Mueller Hinton broth (MBH) in the concentration range of 1−256 μg/mL. Broth was dispensed (200 μL) in the corresponding wells of a 96-well polypropylene microtiter plate. Then, 10 μL of a nutrient broth starter culture of each bacterial strain was added to achieve final inoculums of ca. 5 × 10^−5^ colony forming units (CFU) per mL. Nutrient broth medium without the compound served as growth control. The growth of the microorganisms was determined visually after incubation for 24 h at 37 °C. The development of turbidity in an inoculated medium is a function of growth. A rise in turbidity reflects increases in both mass and cell number. All the experiments were performed in triplicate. Resazurin was used to confirm the visual MIC values. Thus, 20 μL of resazurin at 0.015% *w*/*v* was added to each well and left to react for approximately 30 min at 37 °C. After the incubation period, the blue to pink color change in resazurin was used as indication of bacterial metabolic activity.

#### Antibiofilm Activity

Antibiofilm activity was assessed against biofilm-producing strains of methicillin-resistant *Staphylococcus aureus* (MRSA) ATCC 43300 and *Pseudomonas aeruginosa* PAO1. Biofilm inhibition and eradication capacities of the gemini surfactants were evaluated against adherent bacterial biofilms grown in 96-well microtiter plates [29]. Bacteria were grown overnight in tryptic soy agar at 37 °C for 24 h. The bacteria were suspended in lysogeny broth with glucose (1%) at 1.5 × 10^8^ CFU/mL.

Biofilm inhibition procedure: 100 μL of the gemini surfactant at different concentration values was added to each well in a 96-well microplate, then, 100 μL of the diluted bacterial suspension was added to the wells. Two-fold serial dilutions of the gemini surfactants to be tested were used (2–128 μg/mL). Microtiter plates were incubated at 37 °C for 24 h. Spent growth media was then discarded and biofilm was fixed with 150 μL methanol for 10 min. After the plates were air dried, the biofilms were stained with 50 μL of 0.1 of % crystal violet for 25 min. The plates were rinsed with water and then 150 μL of 96% ethanol containing 10% acetic acid was added to all 96-well plates to dissolve the remaining crystal violet-stained biofilm. The absorbance value was recorded at 570 nm. Each assay was performed at least three times and the results were averaged.

Biofilm eradication procedure: For mature biofilm formation, 200 μL of the diluted bacterial suspension was added to each well in a 96-well microplate and incubated at 37 °C for 24 h. The wells were gently rinsed with phosphate-buffered saline (PBS) and 200 μL of the corresponding gemini surfactant solution was added to each well and incubated again at 37 °C for 24 h. Two-fold serial dilutions of the gemini surfactant were used (4–128 μg/mL). After the plates were air dried, the biofilms were stained with 50 μL of 0.1 of % crystal violet for 25 min. The plates were rinsed with water and then 150 μL of 96% ethanol containing 10% acetic acid was added to all 96-well plates to dissolve the remaining crystal violet-stained biofilm. The absorbance value was recorded at 570 nm. Each assay was conducted at least three times and the results were averaged.

### 3.5. Anti-Enzymatic Activities

#### 3.5.1. Collagenase Inhibitory Activity Assay

The inhibition of collagenase followed the methodology proposed by Thring et al. [47]. First, a solution containing the surfactants at 1000 μg/mL was prepared. An aliquot of 40 μL of each sample was used in the assay. Collagenase from *Clostridium histolyticum* (ChC) was dissolved in tricine buffer (50 mM, pH 8.0) immediately before use at an initial concentration of 0.35 U/mL. The synthetic substrate N-[3- (2-furyl) acryloyl]-Leu-Gly-Pro-Ala (FALGPA) (40 μL) was dissolved in the kit buffer (60 μL) according to the supplier information. The samples of C_3_(LA)_2_, C_6_(LA)_2_ and C_9_(LA)_2_ were incubated with the enzyme for 15 min before the substrate addition. The final reaction mixture contained tricine buffer, 0.8 mM FALGPA, 0.1 U of ChC and approximately 25 μg of each surfactant. Ultrapure water was used as a negative control, while the positive control was EGCG solution, prepared at the same concentration of the surfactants. The absorbance at λ = 345 nm was measured immediately after the substrate incorporation and continuously during 20 min in kinetic mode using an Elx800 Microplate Reader Biotek^®^ (Winooski, VT, USA). The collagenase inhibitory activity of each sample was determined in triplicate, according to the following equation:

(1) $$Collagenase\;inhibitory\;activity\;\left(\%\right)=\frac{A_{1}-A_{2}}{A_{1}}\times 100$$

where *A*_1_ and *A*_2_ represent the absorbance in the absence and presence of sample, respectively.

#### 3.5.2. Elastase Inhibitory Assay

The elastase inhibition was tested according to the method described by Thring et al. [47]. First, a stock solution of each surfactant at 1000 µg/mL was prepared in sterile ultrapure water. A stock solution of porcine pancreatic elastase at 3.33 mg/mL in sterile ultrapure water was also prepared. The substrate N-succinyl-N-succinyl- (Ala) 3-p-nitroanilide was dissolved in 0.2 mM Tris-HCl buffer solution (pH 8.0). The samples (40 µL) were then diluted and incubated together with the enzyme for 15 min prior to the substrate addition. Negative control was sterile ultrapure water, while EGCG (250 μM or 0.114 mg/mL) was used as the positive control. The absorbance at λ = 410 nm was measured immediately after the substrate addition and continuously during the next 2 h using an Elx800 Microplate Reader (Biotek^®^). The elastase inhibition was assessed from the release of p-nitroaniline, revealing the substrate proteolysis and subsequent coloring. The anti-elastase activity (in %), determined in triplicate, was calculated according to the following equation:

(2) $$Elastase\;inhibitory\;activity\;\left(\%\right)=\frac{A_{1}-A_{2}}{A_{1}}\times 100$$

where *A*_1_ and *A*_2_ represent the absorbance in the absence and presence of the testing samples, respectively.

#### 3.5.3. Hyaluronidase Inhibitory Activity

The hyaluronidase inhibitory activity was performed according to the method used by Altinyay et al. [54] with some modifications. Briefly, a work solution containing bovine hyaluronidase (7900 units/mL) in 0.1 M acetate buffer (pH 3.6) was prepared. Fifty microliters of the work solution were mixed 1:1 with the surfactants’ solution in 5% DMSO. For the negative control, 50 μL of 5% DMSO was used, while EGCG was used as positive control. After 20 min of incubation at 37 °C, 50 μL of calcium chloride (12.5 mM) was added to the mixture and again incubated for 20 min at 37 °C. At that point, 250 μL sodium hyaluronate (1.2 mg/mL) was added and incubated for 40 min at 37 °C. After incubation, the mixture was treated with 50 μL of 0.4 M NaOH and 100 μL of 0.2 M sodium borate and then incubated for 3 min in a boiling water bath. After cooling to room temperature, 1.4 mL of p-dimethylaminobenzaldehyde solution was added to the reaction mixture, being further incubated at 37 °C for 20 min. The resulting colored solution was measured at λ = 585 nm (PerkinElmer-Lambda 35, Brazil). The hyaluronidase inhibitory activity of each sample was calculated according to the following formula:

(3) $$Hyaluronidase\;inhibitory\;activity\;\left(\%\right)=\frac{A_{1}-A_{2}}{A_{1}}\times 100$$

where *A*_1_ and *A*_2_ represent the absorbance in the absence and presence of the testing sample, respectively.

### 3.6. Molecular Docking Materials

In order to elucidate the binding modes of the surfactants within enzymes evaluated, a molecular docking simulation was carried out using AutodockVina [55]. The crystal structure of the collagenase unit of a Vibrio collagenase from Vibrio harveyi VHJR7 (PDB ID: 7ESI), 3D structures of porcine pancreatic elastase (PDB ID: 1ELE) and crystal structure of bee venom hyaluronidase (PDB ID: 1FCV) were selected. Polar hydrogen atoms have been added to the protein’s structures for correcting ionization and tautomeric states of amino acid residues [56]. The binding sites on the structure of collagenase, elastase and hyaluronidase were made using the Auto Grid step using the original ligands as a reference. The grid box with number points in the box (x, y, z) (16, 38, 16) has been chosen. All parameters were default settings using Autodock Tools 1.5.4 44. The surfactants and EGCG ligands were drawn using Chemdraw20.1.1 software v.1. To select the most stable conformation, the geometry of ligands was subsequently optimized using Molecular Force Field (MMFF94). The ligand and target protein files were converted to the PDBQT format to make it suitable for docking in AutoDock Vina. The interactions of complex protein–ligand conformations were analyzed by Discovery Studio Client software 22.1.

### 3.7. Cell Culture

The immortal human keratinocyte (HaCaT) and the squamous cell carcinoma (A431) were obtained from Celltec, University of Barcelona. The cells were grown in DMEM medium (4.5 g/L glucose) supplemented with 10% (*v*/*v*) FBS, 2 mM L-glutamine, 100 U/mL of penicillin and 100 μg/mL of streptomycin at 37 °C under 5% CO_2_. Cells were routinely cultured in 75 cm^2^ culture flasks and were trypsinized using trypsin-EDTA when reached approximately 80% of confluence.

#### 3.7.1. Cell Viability Assays

HaCaT cells (1 × 10^5^ cells/mL) and A431 cells (5 × 10^4^ cells/mL) were grown at the defined densities into the central 60 wells of a 96-well microplate. Cells were incubated for 24 h under 5% CO_2_ at 37 °C. The medium was then removed and the cells were incubated with the surfactants (100 μL), previously diluted 1:1 (*v*/*v*), reaching a concentration of 17.8 µg/mL in DMEM medium supplemented with 5% FBS using two different colorimetric methods for quantification, as follows.

#### 3.7.2. NRU Assay

The accumulation of the neutral red dye in the lysosomes of viable, undamaged cells constitutes the basis of the neutral red uptake (NRU) assay. After the cells were incubated for 24 h with the corresponding systems at 35.6 µg/mL, the medium was removed and the surfactants´ solutions were incubated for 3 h with the NRU dye solution (50 μg/mL) dissolved in the medium without FBS and phenol red (Lonza). Cells were then washed with sterile PBS, followed by the addition of 100 μL of a solution containing 50% ethanol absolute and 1% acetic acid in distilled water to extract the non-adhered dye. The microplates were then stirred for 5 min at 25 °C to promote the dissolution. Finally, the absorbance of the resulting solutions was measured at λ = 550 nm using a Bio-Rad 550 microplate reader (Hercules, CA, USA).

#### 3.7.3. MTT Assay

This method is based on the living cell’s capability of reducing the yellow tetrazolium salt, 2,5 Diphenyl-3, -(4,5-dimethyl-2-thiazolyl) tetrazolium bromide (MTT) to insoluble purple formazan crystals. After the cells were incubated for 24 h with the surfactants’ solutions or the surfactant-loaded nanoparticles (at 35.6 µg/mL), the medium was removed and 100 μL of MTT in PBS (5 mg/mL) diluted 1:10 in culture medium (without phenol red and FBS) were added to the cells. The plates were incubated for 3 h and the medium was immediately removed thereafter. After that, 100 μL of DMSO was added to each well to dissolve the purple formazan crystals. Finally, the absorbance of the resulting solutions was measured at 550 nm using a Bio-Rad 550 microplate reader (Hercules, CA, USA).

### 3.8. Statistical Analysis

The results were expressed as mean ± SD. The data were analyzed using ANOVA followed by Tukey’s post-test. A value of *p* < 0.05 was considered statistically significant. Statistical analyses were performed using the software Graphpad Prism version 7.0.

## 4. Conclusions

Biodegradable gemini cationic surfactants based on the arginine amino acid show good antimicrobial activity against Gram-positive and Gram-negative bacteria, including some problematic resistant microorganisms such as *MRSA* and *P. aeruginosa*. These gemini surfactants are more active against the Gram-positive bacteria; moreover, they have a stronger efficiency as antimicrobial agents than their corresponding single-chain homologues. The antimicrobial efficiency of these surfactants depends on their hydrophobic alkyl chain length and their spacer chain nature. The highest antibacterial activity was observed for the surfactant bearing a C_10_ hydrophobic group and a C_3_ spacer chain. In addition to having very good antibacterial activity, these arginine-based surfactants can prevent the growth of biofilms and eradicate them once formed. The best antibiofilm activity was obtained for gemini surfactants with spacer chains containing three methylene groups and C_10_–C_12_ alkyl chains. These surfactants exhibited some elastase inhibition and moderate anti-collagenase activity; however, they increased the enzymatic activity of the hyaluronidase. Considering the NRU method, the C_3_ and C_6_ exhibit low cytotoxicity at 35.6 µg/mL; a concentration higher than the MIC values obtained for these surfactants against most of the tested microorganisms.

The obtained results make these surfactants promising antimicrobial compounds to be used in biomedical applications. Moreover, their anti-enzymatic activity suggests that they can be used in the treatment of specific skin disorders.

## Figures and Tables

**Table 1:** Apparent pKa values and CMC of the arginine-based surfactants.

Compound	Length of Spacer Chain	pKap	CMC (mM) Conductivity	CMC (mM) Fluorescence	CMC (mM) Surface Tension	γ_cmc_ mN/m
C3(OA)2	3	n.d.	n.d.	8 *	n.d.	n.d.
C3(CA)2	3	n.d.	2 *	3 *	0.043 *	32 *
C_3_ (LA)_2_ OH	3	10.9	0.6 *	0.6 *	n.d.	n.d.
C_3_ (LA)_2_	3	11.4	0.5 *	0.4 *	0.005 *	35 *
C_6_ (LA)_2_	6	10.5	0.4 *	0.4 *	0.002 *	30 *
C_9_ (LA)_2_	9	9.4	0.3 *	0.3 *	0.003 *	34 *

* Results from reference [17]. n.d. (non determined).

**Table 2:** Results of the interaction details and docking score in (kcal/mol) of the C_3_(LA)_2_, C_6_(LA)_2_, C_9_(LA)_2_ and EGCG ligands in the active sites on the surface of Collagenase (PDB ID: 7ESI).

	Compounds	Docking Score (kcal/mol)	Ligand	Receptor Pocket	Interaction Types	Distance (Å)
**Collagenase**	C_3_(LA)_2_	−12	O(C=O)	GLU254	Hydrogen Bond	2.56398
			O(C=O)	ASN253	Hydrogen Bond	3.24822
			H(CH2)	TRP252	Hydrogen Bond	3.45238
	C_6_(LA)_2_	−10	N(NH)	GLU159	Electrostatic	4.30283
			H(NH)	GLU1	Hydrogen Bond	2.05525
			H(NH Guanidine)	SER208	Hydrogen Bond	2.24523
			H(NH Guanidine)	GLU159	Hydrogen Bond	1.97905
	C_9_(LA)_2_	−11.8	N(NH)	GLU201	Electrostatic	4.75159
			H(NH)	SER208	Hydrogen Bond	2.39595
			CH3	TRP234	Hydrophobic	4.32331
			CH3	TRP234	Hydrophobic	4.92808
			CH3	TYR235	Hydrophobic	5.06206
	EGCG	−10.9	O(OH)	SER423	Hydrogen Bond	2.78691
			O(ROR)	THR438	Hydrogen Bond	2.99005
			H(OH)	THR7	Hydrogen Bond	2.20889
			Ring	GLU8	Hydrogen Bond	2.3916
			O(ROR)	GLY427	Hydrogen Bond	3.02574
			Ring	ASP424	Electrostatic	4.72785
			Ring	GLU1	Electrostatic	4.21691

**Table 3:** Results of the interaction details and docking score in (kcal/mol) of the C_3_(LA)_2_, C_6_(LA)_2_, C_9_(LA)_2_ and EGCG ligands in the active sites on the surface of Elastase (PDB ID: 1ELE).

	Compounds	Docking Score (kcal/mol)	Ligand	Receptor Pocket	Interaction Types	Distance (Å)
**Elastase**	C_3_(LA)_2_	−12.8	H(NH)	ASP63	Electrostatic	4.22808
			H(NH Guanidine)	HIS60	Hydrogen Bond	2.64398
			CH3	LEU156	Hydrophobic	3.95092
	C_6_(LA)_2_	−10.9	N(NH)	ASP63	Electrostatic	4.06755
			O(C=O)	THR182	Hydrogen Bond	2.79448
			O(C=O)	VAL224	Hydrogen Bond	2.72013
			H(NH)	HIS60	Hydrogen Bond	3.00088
			H(NH Guanidine)	HIS60	Hydrogen Bond	1.92249
			CH3	LEU66	Hydrophobic	5.08936
			CH3	TYR35	Hydrophobic	4.15468
	C9C12	−9.9	O(C=O)	HIS60	Hydrogen Bond	2.67117
			O(C=O)	THR182	Hydrogen Bond	2.12153
			H(NH Guanidine)	THR182	Hydrogen Bond	2.91942
			CH3	LEU66	Hydrophobic	4.47598
			CH3	TYR35	Hydrophobic	5.20529
	EGCG	−9.2	H(OH)	HIS60	Hydrogen Bond	2.25921
			O(C=O)	SER203	Hydrogen Bond	2.43056
			O(OH)	VAL224	Hydrogen Bond	2.01638
			O(C=O)	HIS60	Hydrogen Bond	2.79502
			H(OH)	THR44	Hydrogen Bond	2.19728
			Ring	HIS60	Electrostatic	4.57072
			Ring	CYS45	Other	5.93844
			Ring	HIS60	Hydrophobic	4.66195

**Table 4:** Results of the interaction details and docking score in (kcal/mol) of the C_3_(LA)_2_, C_6_(LA)_2_, C_9_(LA)_2_ and EGCG ligands in the active sites on the surface of Hyaluronidase (PDB ID: 1FCV).

	Compounds	Docking Score (kcal/mol)	Ligand	Receptor Pocket	Interaction Types	Distance (Å)
**Hyaluronidase**	C_3_(LA)_2_	−12.3	N(NH)	GLU113	Electrostatic	3.80065
			O(C=O)	ARG47	Hydrogen Bond	2.30683
			O(C=O)	GLU113	Hydrogen Bond	2.79755
			CH3	HIS23	Hydrophobic	5.49822
	C_6_(LA)_2_	−11.8	H(NH)	GLU113	Hydrogen Bond; Electrostatic	2.79842
			O(C=O)	ARG47	Hydrogen Bond	2.20476
			O(C=O)	ARG47	Hydrogen Bond	2.69873
			O(C=O)	SER304	Hydrogen Bond	1.76159
			H(NH Guanidine)	GLN271	Hydrogen Bond	2.82288
	C_9_(LA)_2_	−12	H(NH)	GLU113	Electrostatic	4.05683
			O(C=O)	ASN97	Hydrogen Bond	2.52287
			O(C=O)	ASN97	Hydrogen Bond	2.56576
			O(C=O)	GLU113	Hydrogen Bond	2.62381
			H(NH Guanidine)	TYR184	Hydrogen Bond	2.78541
			H(NH Guanidine)	GLN271	Hydrogen Bond	2.40937
	EGCG	−9.7	O(C=O)	THR193	Hydrogen Bond	2.97624
			O(C-O-C)	THR193	Hydrogen Bond	2.60255
			O(OH)	GLN271	Hydrogen Bond	2.60537
			O(OH)	EU192	Hydrogen Bond	2.37614
			O(C=O)	TYR190	Hydrogen Bond	3.51835
			O(OH)	SER225	Hydrogen Bond	3.6589
			Ring	ARG229	Electrostatic	4.45853
			Ring	ARG244	Electrostatic	3.70196
			Ring	PRO194	Hydrophobic	5.22577

## Data Availability

Not applicable.

## Sample Availability

Sample of all the compounds are available from the authors.
